# Dietary-phytochemical mediated reversion of cancer-specific splicing inhibits Warburg effect in head and neck cancer

**DOI:** 10.1186/s12885-019-6257-1

**Published:** 2019-11-01

**Authors:** Sandhya Yadav, Somnath D. Bhagat, Amit Gupta, Atul Samaiya, Aasheesh Srivastava, Sanjeev Shukla

**Affiliations:** 1 Dept of Biological Sciences, Indian Institute of Science Education and Research Bhopal, Bhopal Bypass Road, Bhauri, Bhopal, Madhya Pradesh 462066 India; 2 Dept of Chemistry, Indian Institute of Science Education and Research Bhopal, Bhopal Bypass Road, Bhauri, Bhopal, Madhya Pradesh 462066 India; 3 Bansal Hospital, Bhopal, Madhya Pradesh 462016 India

**Keywords:** Curcumin, Head and neck cancer, PKM, Warburg effect, Alternative splicing

## Abstract

**Background:**

The deregulated alternative splicing of key glycolytic enzyme, Pyruvate Kinase muscle isoenzyme (PKM) is implicated in metabolic adaptation of cancer cells. The splicing switch from normal PKM1 to cancer-specific PKM2 isoform allows the cancer cells to meet their energy and biosynthetic demands, thereby facilitating the cancer cells growth. We have investigated the largely unexplored epigenetic mechanism of PKM splicing switch in head and neck cancer (HNC) cells. Considering the reversible nature of epigenetic marks, we have also examined the utility of dietary-phytochemical in reverting the splicing switch from PKM2 to PKM1 isoform and thereby inhibition of HNC tumorigenesis.

**Methods:**

We present HNC-patients samples, showing the splicing-switch from PKM1-isoform to PKM2-isoform analyzed via immunoblotting and qRT-PCR. We performed methylated-DNA-immunoprecipitation to examine the DNA methylation level and chromatin-immunoprecipitation to assess the BORIS (Brother of Regulator of Imprinted Sites) recruitment and polII enrichment. The effect of dietary-phytochemical on the activity of denovo-DNA-methyltransferase-3b (DNMT3B) was detected by DNA-methyltransferase-activity assay. We also analyzed the Warburg effect and growth inhibition using lactate, glucose uptake assay, invasion assay, cell proliferation, and apoptosis assay. The global change in transcriptome upon dietary-phytochemical treatment was assayed using Human Transcriptome Array 2.0 (HTA2.0).

**Results:**

Here, we report the role of DNA-methylation mediated recruitment of the BORIS at exon-10 of *PKM*-gene regulating the alternative-splicing to generate the PKM2-splice-isoform in HNC. Notably, the reversal of Warburg effect was achieved by employing a dietary-phytochemical, which inhibits the DNMT3B, resulting in the reduced DNA-methylation at exon-10 and hence, *PKM*-splicing switch from cancer-specific PKM2 to normal PKM1. Global-transcriptome-analysis of dietary-phytochemical-treated cells revealed its effect on alternative splicing of various genes involved in HNC.

**Conclusion:**

This study identifies the epigenetic mechanism of *PKM*-splicing switch in HNC and reports the role of dietary-phytochemical in reverting the splicing switch from cancer-specific PKM2 to normal PKM1-isoform and hence the reduced Warburg effect and growth inhibition of HNC. We envisage that this approach can provide an effective way to modulate cancer-specific-splicing and thereby aid in the treatment of HNC.

## Background

Cancer cell compensates the energy requirement by rewiring its metabolism so as to promote the proliferation and survival [1]. The aerobic glycolysis or Warburg effect coupled with increased glucose-uptake and lactate-production is the most important and almost universally implicated in providing the growth advantage to the cancer cells [2, 3]. The Pyruvate kinase M catalyzes one of the rate-limiting steps of glycolysis and the cancer-specific spliced isoform of Pyruvate kinase, PKM2 is known to promote the Warburg effect and therefore facilitates the tumor growth [4, 5]. The PKM has two spliced isoforms: the alternative inclusion of mutually exclusive exon 9 and exon 10 leads to the generation of PKM1 and PKM2 isoform respectively. The PKM1 isoform is expressed in the normal cells [4] and is associated with normal glucose metabolism wherein PKM2 isoform is overexpressed in cancer cells [5] and is associated with increased aerobic glycolysis, termed as Warburg effect, which is associated with the increased cell proliferation and reduced apoptosis [6], thereby PKM2 may be a potential therapeutic target for cancer treatment [7]. Therefore, it becomes important to understand the mechanism of splicing switch from PKM1 to PKM2 in cancer cells.

The Warburg effect is significantly upregulated in HNC [8], which is the 6th most common cancer worldwide with an incidence of 650,000 new cases every year [8] and more than 350,000 deaths every year [9]. Although the PKM2 overexpression is reported in HNC where it is associated with the poor prognosis [10, 11], the mechanism of regulation of PKM alternative splicing has not been studied in head and neck cancer.

PKM1 downregulation (exon 9 exclusion) is reported to be mediated by the members of the hnRNP family (heterogeneous nuclear ribonucleoprotein) hnRNPA1, hnRNPA2, and PTB (Polypyrimidine tract-binding protein 1) [12]. These hnRNPs are upregulated by the oncogene *MYC* and are reported to promote exon 9 exclusion by binding to exon 9 flanking sequences [12]. Additionally, the splicing activators SR family protein SRSF3 has also been shown to affect the inclusion of exon 10 [13]. Recently, we have shown that the *PKM* splicing switch is regulated epigenetically by DNA methylation-dependent binding of BORIS at exon 10 of *PKM* gene leading to the inclusion of exon 10 to generate the PKM2 splice isoform in breast cancer [14]. Studies have shown that the increased expression of PKM2 in various cancers including HNC is correlated with cancer progression [10, 15]. However, the underlying epigenetic mechanism leading to the splicing switch of PKM1 to PKM2 remains to be established in HNC.

Interestingly, the epigenetic modifications involved in cancer progression are potentially reversible [16–18]. Thus, the epigenetic mechanism regulating the *PKM* splicing can be targeted to revert the cancer-specific isoform to normal splice isoform. Curcumin, the active component of the herb *Curcuma longa*, has recently been shown to decrease the Warburg effect in cancer cells by reducing the PKM2 level [19]. Curcumin is a dietary polyphenolic compound enriched in the roots of turmeric with a broad therapeutic potential for cancer [20]. Curcumin shows antitumor activity in colorectal cancer cells [21] and plays an anti-leukemic role in acute myeloid leukemia [22]. Curcumin has also been proposed to be effective against cancer progression by inducing apoptosis [23]. Additionally it also affects the key pathways which regulate cell survival [23], proliferation [24], metastasis [25], and angiogenesis [26]. Considering the observed role of curcumin on Warburg effect [19], we investigated whether the curcumin reverts the Warburg effect by regulating the *PKM* splicing through epigenetic alterations.

Here in this study, we present the underlying epigenetic mechanism of *PKM* splicing switch in HNC patients samples as well as provide the first mechanistic evidence of intragenic DNA demethylation ability of curcumin by which curcumin reverts the *PKM* splicing from cancer-specific PKM2 isoform to PKM1 isoform in HNC.

## Materials and methods

### Cell culture

The two cell lines used in this study, H157 [squamous cell carcinoma (SCC) of the buccal mucosa of a male patient, age 84] and H413 [squamous cell carcinoma (SCC) of the buccal mucosa of a 53 year-old female patient] were obtained from European Collection of Authenticated Cell Culture (ECACC) (Salisbury UK) in May 2014. The HNC cell lines H157 cell (ECACC 07030901) and H413 cell (ECACC 06092007) were cultured in ECACC recommended growth medium (1:1 ratio of DMEM (Gibco, 11,995–065) and Ham’s F-12 (Gibco, 11,765–054) supplemented with 10% Fetal Bovine Serum (Invitrogen, 16,000,044) and 2 mmol L-glutamine (Sigma, G7513) at 37 °C with 5% CO_2_. Both the cell lines were authenticated in May 2019 by STR analysis and were regularly tested for mycoplasma contamination.

### Head and neck cancer sample collection

Tumor and adjacent normal tissue pairs were collected from patients undergoing surgery for HNC at Bansal Hospital, Bhopal, India. The tissue samples were immediately snap-frozen in liquid nitrogen after surgery and stored at − 80 °C until use. One part of the tumor and adjacent normal tissue pairs were kept in RNA later (Thermo Fisher Scientific, AM7024) for RNA isolation after surgery, snap frozen and stored at − 80 °C until use. The study was approved by Ethics Committee of the Indian Institute of Science Education and Research Bhopal. The informed consent forms were obtained from all the patients. Details of the patients used in the study are presented in Table 1.

**Table 1:** Clinical characteristics of patients

S.No.	Patient	Histopathology
1	Patient 1	Carcinoma tongue
2	Patient 2	Left buccal mucosa
3	Patient 3	Right lateral border of tongue
4	Patient 4	Right lower GBS with bone erosion (on CT)
5	Patient 5	Left lower Gingivo-buccal sulcus
6	Patient 6	Buccal mucosa
7	Patient 7	Left lateral border of tongue
8	Patient 8	Right buccal mucosa
9	Patient 9	Tongue
10	Patient 10	Right buccal mucosa with carcinoma left
11	Patient 11	Left buccal mucosa

### Curcumin treatment

Curcumin loaded polyelectrolyte complexes (Curcumin-PECs) was prepared, as reported previously [27]. It was used for the treatment, while the control consisted of the PECs without Curcumin. HNC Cells were cultured in DMEM: F12 (1:1) containing 10% FBS, L-glutamine. After 24 h of seeding, cells were serum-starved for 6-8 h before treatment with Curcumin-PECs and the treatment was repeated after every 24 h. The cells were harvested at the fourth day of cell seeding, and the total RNA was extracted, cDNA was prepared, and qRT-PCR was performed to check the effect of curcumin on genes and the exons of interest. Similarly, the cells were treated with 10 μM 5-Aza-2′-deoxycytidine (Sigma, 83,656) for 48 h and RNA was extracted at the third day of cell seeding.

### Cell viability assay

Cells (15.6 × 10^3^ cells/cm^2^) were seeded in 6-well culture plates for 24 h at 37 °C with 5% CO_2_. Cells were then treated with (10 μM, 25 μM, 50 μM, 75 μM) different concentrations of the curcumin-PEC and the PEC control. After treatment, the cells were harvested and diluted with an equal volume of 0.4% trypan blue. The populations of live and dead cells were counted using hemocytometer, under the microscope.

Cell viability was calculated using the formula**:**

$$ \%\mathrm{cell}\ \mathrm{viability}=\left[\mathrm{Live}\ \mathrm{cells}/\left(\mathrm{Live}\ \mathrm{cells}+\mathrm{Dead}\ \mathrm{cells}\right)\right]\ \mathrm{X}\ 100 $$

### Curcumin uptake assay

The H157 cells were treated with different concentrations (2.5 μM, 5 μM, 10 μM) of curcumin-PEC and free-curcumin and incubated for 4 h at 37 °C with 5%CO_2_. Post-incubation cells were washed with 1X PBS and fixed with 3.4% formaldehyde (Sigma F8775). The formaldehyde-fixed cells were stained with the DAPI (4′,6-Diamidine-2′-phenylindole dihydrochloride) (Invitrogen D1306) for 10 min and the auto-fluorescence (GFP: Green fluorescent protein) of curcumin overlapping with DAPI fluorescence was imaged at 40x magnification under microscope.

### Nuclear protein isolation

Nuclear protein isolation from H157 cells was performed by following the methodology as described. Briefly, the cell pellet was collected and resuspended in hypotonic buffer (20 mM Tris-HCl, pH 7.4, 10 mM NaCl, 3 mM MgCl_2_) and incubated for 15 min on ice. Post-incubation, cells were centrifuged at 3000 rpm at 4 °C for 10 min to pellet the nuclei. The nuclei pellet was lysed with extraction buffer (10 mM Tris, pH 7.4,2 mM Na_3_VO_4,_100 mM NaCl,1% Triton X-100,1 mM EDTA,10% glycerol,1 mM EGTA, 0.1% SDS,1 mM NaF,0.5% deoxycholate, 20 mM Na_4_P_2_O_7_) by centrifugation at 14000 g at 4 °C for 30 min. and the nuclear fraction was collected in the supernatant.

### RNA interference

The H157 HNC cells were infected with lentivirus containing small hairpin RNA (shRNA) purchased from Sigma (Saint Louis, USA) specific to DNMT1(shDNMT1), DNMT3A (shDNMT3A), DNMT3B (shDNMT3B) and eGFP (shControl) using 8 μg/ml polybrene containing media. Cells were selected with 1 μg/ml puromycin for 2 days. Post selection cells were used for downstream experiments.

### Oligo sequence of shRNAs

**Table:** 

eGFPshControl	5′-CCGGTACAACAGCCACAACGTCTATCTCGAGATAGACGTTGTGGCTGTTGTATTTTT-3′
shDNMT3B_1	5′-CCGGCCATGCAACGATCTCTCAAATCTCGAGATTTGAGAGATCGTTGCATGGTTTTTG-3’
shDNMT3B_2	5′-CCGGCCATGCAACGATCTCTCAAATCTCGAGATTTGAGAGATCGTTGCATGGTTTTTG-3’
shDNMT1_1	5′-CCGGCGACTACATCAAAGGCAGCAACTCGAGTTGCTGCCTTTGATGTAGTCGTTTTT-3’
shDNMT1_2	5′-CCGGGCCGAATACATTCTGATGGATCTCGAGATCCATCAGAATGTATTCGGCTTTTT-3’
shDNMT3A_1	5′-CCGGCCACCAGAAGAAGAGAAGAATCTCGAGATTCTTCTCTTCTTCTGGTGGTTTTTG-3’
shDNMT3A_2	5′-CCGGCCGGCTCTTCTTTGAGTTCTACTCGAGTAGAACTCAAAGAAGAGCCGGTTT TTG-3’

### DNA methyltransferase activity assay

The DNA methyltransferase activity was performed using the DNMT activity quantification kit (Abnova, KA1547) as per the manufacturer’s protocol. Briefly, nuclear protein extract of the H157 cells and pure DNMT3B enzyme (Abcam,ab170410) were treated with curcumin in vitro, and the effect of curcumin over methyltransferase activity was quantified based on color intensity.

### Quantitative RT-PCR

Total RNA was isolated using Trizol (Ambion, 15,596,018) from cultured H157 and H413 cells (HNC cells) and HNC patients samples according to the manufacturer’s instruction. RNA was quantified using Nanodrop (Thermo Fisher Scientific, ND8000) and 1 μg of RNA was reverse transcribed by iScript complementary DNA (cDNA) synthesis kit (BioRad, 17,088) as per the manufacturer’s instructions. The amplification reaction was performed using SYBR green (Affymetrix, 75,665) with light cycler 480 II (Roche) according to manufacturer’s instruction. The primers used in this study were designed using IDT PrimerQuest tool (https://www.idtdna.com/) and are mentioned in Table 2. The average cycle thresholds of three independent experiments were calculated and then normalized to housekeeping control gene RPS16 using the formula: [2^^(Ct control – Ct target)^]. In addition, constitutive exon normalization was performed for exon-level expression analysis. Student’s t-test was used to compare gene/exon expression between two different groups and *P* < 0.05 was considered as statistically significant.

**Table 2:** List of primer sequences utilized for qRT-PCR

S.No.	Primers	Sequence
1	PKM E11 Fw	CCATCATTGCTGTGACCCGGAAT
2	PKM E11 Rev	CATTCATGGCAAAGTTCACCCGGA
3	PKM Ex10 Fw	TAGATTGCCCGTGAGGCAGAGGCT
4	PKM Ex10 Rev	TGCCAGACTTGGTGAGGACGATTA
5	PKM Ex8–9 Fw	ATGCAGCACCTGATAGCTCGTGA
6	PKM Ex9 Fw	GTTCCACCGCAAGCTGTTTGAAGA
7	PKM Ex9 Rev	TGCCAGACTCCGTCAGAACTATCA
8	PKM E10–11 Fw	TCACCAAGTCTGGCAGGTCTG
9	RPS16 SET5 Fw	AAACGCGGCAATGGTCTCATCAAG
10	RPS16 SET5 Rev.	TGGAGATGGACTGACGGATAGCAT
11	DNMT3A EX7 Fw	GCCAAGGTCATTGCAGGAA
12	DNMT3A EX7 Rev	CGTACTCTGGCTCGTCATC
13	DNMT3B EX5 Fw	AACAGCATCGGCAGGAA
14	DNMT3B EX5 Rev	GATACTCTGAACTGTCTCCATCTC
15	DNMT1 EX4 Fw	TGCTTACAACCGGGAAGTGAATGG
16	DNMT1 EX4 Rev	TTGGCATCTGCCATTCCCACTCTA
17	TBC1D4 E7 Fw	CAGTGACCAGGAAGAAAATGAAC
18	TBC1D4 E7 Rev	CACGTGTGTCTTCTGCTTGG
19	TBC1D4 E8 Fw	AATAGTACAATCCCAGAAAATGCAA
20	TBC1D4 E8 Rev	CCTTGAGAAGATATTTTCCAGGG
21	TBC1D4 Cons Fw	AGAGCCAAGCTGGTGATACAG
22	TBC1D4 Cons Rev	CTGAACTCTTTCAAAGATGTCAGC
23	TBC1D4 Ex7–8 Rev	TATTTGAAATAGTAGAAGGGCCTTCC
24	VPS39 Ex 2–3 Fw	CGGAAGGACGTTGTGCCAGCAGAT
25	VPS39 Ex3 Rev	TTGCAACTGCCGCTTTCAGGT
26	VPS39 Ex4 Fw	ATCTATGTGGCCAGCAATCA
27	VPS39 Ex4 Rev	GCTGCAGAGCCAATTCAAAC
28	VPS39 Ex3 Fw	CCTGTATTTGGAACTACCAGTGT
29	VPS39 10 Fw	ATCTATGTGGCCAGCAATCA
30	VPS39 10 Rev	GCTGCAGAGCCAATTCAAAC
31	ZNF207 Ex8–9 Fw	AGTGCTGGACAGATGGGGACAC
32	ZNF207 Ex 9 Fw	TTTGACCCATTTGTTTGGAG
32	ZNF207 Ex9 Rev	TTGTGCTGTGCTAGGAAACAGAG
33	ZNF207 Ex8 Fw	GATGCCGTACCAAATGCAATAC
34	ZNF207 Ex8 Rev	TCGTCGTCTTTCATCCATGTC

### Immunoblotting

The proteins were separated by sodium dodecyl sulfate-polyacrylamide gel electrophoresis (SDS-PAGE) and transferred to polyvinylidene difluoride (PVDF) membrane (Millipore). The protein-containing PVDF membranes were then probed with following primary antibodies: Anti- PKM1 (Cell Signaling Technology,7067S), Anti-PKM2 (Cell Signaling Technology, 4053S) to identify the level of PKM isoform, Anti- BORIS (Millipore ABE631), AntiDNMT3B (Abcam, ab13604), anti-flag (Novus Biologicals, NBP1-06712SS) and Anti GAPDH (Cell Signaling Technology, 5174S) were used as loading controls for protein assays. After 2 h incubation with primary antibody at room temperature (RT), membranes were washed with 1X tris-buffered saline and Tween-20 (TBST) then again incubated with secondary antibodies for 45 min at RT. The probed PVDF membranes were washed, and the bands were visualized using an Odyssey membrane Scanning system (Li-Cor Biosciences, Bad Homburg, Germany).

### Methylated DNA immunoprecipitation (MeDIP)

Genomic DNA was isolated using Trizol (Ambion, 15,596,018) from H157 cell line and HNC patient’s tissue and MeDIP assay were performed as per the protocol described previously [14]. Briefly, genomic DNA was sonicated, and 3 μg of the sonicated genomic DNA was incubated with 5-Methyl cytosine antibody (Active Motif, 39,649) and Normal mouse IgG antibody (Calbiochem NI03) for overnight at 4 °C. 5% input and Immunoprecipitated fractions were analyzed by qRT-PCR in duplicate using the SYBR Green master mix (Affymetrix, 75,665) and specific primers (table-2) across the exonic regions. Normalization was performed with input using the formula: [2^(Ct input – CT immunoprecipitation)]. Resultant values were further normalized relative to the mouse Ig control I*P* values for the primer set. Student’s t-test was used to identify the significance between two different groups. *P* < 0.05 was considered statistically significant.

### Chromatin immunoprecipitation (ChIP)

ChIP assays were performed as described previously [14]. Briefly, the chromatin was sonicated, and 25 μg of chromatin was immunoprecipitated using the antibody of interest followed by overnight incubation at 4 °C. The following antibodies were used for ChIP: Anti-BORIS (Millipore ABE631), Anti- RNA Pol II (Millipore 1,710,044), Normal Rabbit IgG (Millipore 12,370), Normal mouse IgG (Calbiochem NI03). Immunoprecipitated fractions and 5% input were analyzed by quantitative real-time PCR in duplicate using the SYBR Green Master Mix (Affymetrix, 75,665) and specific primers (table-2) across the exonic regions.

### Lactate assay

The H157 cells (3X10^5^) were transfected with PKM2 overexpression plasmid [14] using Lipofectamine reagent (Thermo Fisher Scientific, L3000–008) as per the manufacturer’s instructions and after 48 h PKM2 overexpressed cells, as well as vector control cells, were treated with curcumin-PEC and PEC-control in six-well culture plates. An equal number of cells were homogenized in the presence of lactate assay buffer and centrifuged at 13,000 g for 10 min. Lactate quantification was performed using a commercially available lactate assay kit (Sigma, MAK064) in a 96-well plate as per the manufacturer’s instruction. Lactate level was measured with a plate reader at an optical density of 570 nm.

### Glucose uptake assay

The H157 cells (3X10^5^) were transfected with PKM2 overexpression plasmid, and after 48 h PKM2 overexpressed cells were treated with curcumin-PEC and PEC-control in six-well culture plates. An equal number of cells were homogenized in the presence of glucose assay buffer and centrifuged at 13,000 g for 10 min. Glucose level quantification was performed using a commercially available glucose assay kit (Abcam ab65333) in a 96-well plate as per the manufacturer’s instruction. Glucose level was measured with a plate reader at an optical density of 570 nm.

### Caspase 3/7 assay

The H157 cells (3X10^5^) were transfected with PKM2 overexpression plasmid, and after 48 h cells were trypsinized and seeded in 96 well plates. After 24 h cells plated in 96 well plates including PKM2 overexpressed cells were treated with curcumin-PEC. After 48 h, Caspase 3/7 activation in cells with curcumin-PEC treatment after PKM2 overexpression and its vector control, as well as curcumin-PEC treated, using the Caspase- 3/7 assay (Promega, G8090) as recommended by the manufacturer. Luminescence readings were taken using a Glomax multi-detection system.

### Invasion assay

The H157 cells (3X10^5^) were transfected with PKM2 over-expression plasmid, and after 48 h PKM2 overexpressed cells were treated with curcumin-PEC and PEC-control in 6 well culture plates. An equal number of cells were seeded in 12-well trans-well insert filters for invasion assay. After 36 h incubation at 37 °C in a CO_2_ incubator, the membranes were collected and stained with crystal violet. The number of cells that migrated to the undersurface of the membrane was examined under a microscope, photographed. Randomly selected microscopic fields from three independent wells were counted using image-j.

### Human Transcriptome Array (HTA) 2.0 data analysis

Total RNA samples were isolated from control-PEC, and curcumin-PEC treated cells, and Affymetrix GeneChip Human Transcriptome Array 2.0 (HTA2.0) kit (Gene Chip® kit cat. no. 900720) protocol was used for the HTA2.0 array profiling. The raw HTA 2.0 array files were normalized by SST-RMA method using Expression Console software and analyzed for the global alternative splicing analysis using Transcriptome Array Console. Splicing Index (SI) was set as the criteria for exon inclusion and exclusion levels in alternative splicing analysis, and it was defined as the ratio of normalized exon intensity (NI) under two conditions. The SI was calculated using the following formula, (https://tools.thermofisher.com/content/sfs/brochures/id_altsplicingevents_technote.pdf);

$$ \mathrm{Splicing}\ \mathrm{Index}\ \left(\mathrm{SI}\right)=\mathrm{log}2\ \left(\mathrm{Sample}\ 1\ \mathrm{NI}/\mathrm{Sample}\ 2\ \mathrm{NI}\right) $$

The positive SI means inclusion, whereas negative SI means exclusion.

The splicing index (linear) ≤ − 2 or splicing index (linear) ≥ + 2 with the *P* < 0.05 criteria were set to measure the pattern of alternatively spliced genes. The heat map was prepared through Morpheus, an online tool provided by Broad Institute (https://software.broadinstitute.org/morpheus/). Gene Ontology analysis of alternatively spliced genes was performed to identify the top GO functions regulated by curcumin-treated H157 cells in molecular and biological process category.

### Statistical analysis

Statistical analysis was performed using GraphPad Prism5 (La Jolla, CA, USA). In the bar graph, unpaired two-tailed Student’s *t*-test was used to compare the differences between two groups. The differences were considered as statistically significant with *P < 0.05, **P < 0.01 and ***P < 0.001, non-significant (ns) difference (*P* > 0.05).

## Results

### *PKM* splicing and it’s correlation with BORIS and RNA pol II enrichment in HNC patients samples

**Fig. 1:**
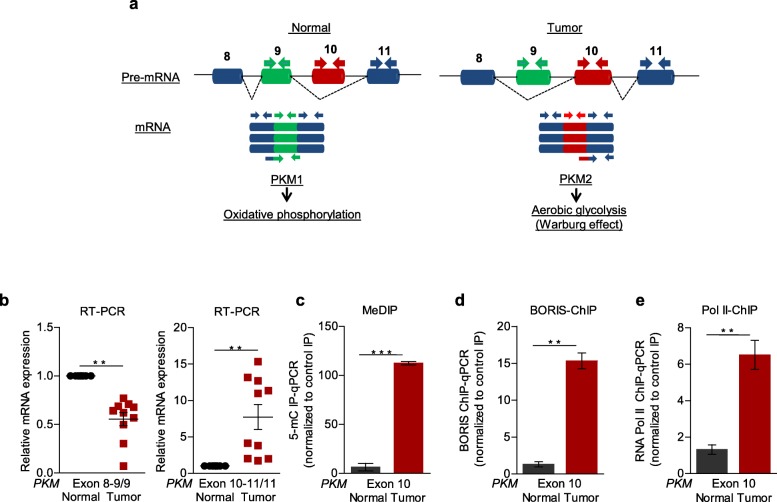
Clinical relevance of PKM splicing and it’s correlation with BORIS. **(a)** Schematic representation of PKM spliced isoform, the cancer-specific PKM2 isoform containing exon 9 whereas normal PKM1 isoform contains exon 10 as has been represented in the processed mRNA with the primer sets directed against the specific exon as a whole, exon junction-specific primer sets were used to specifically measure the spliced isoforms. The exon 9 inclusion was indicated by the exon junction primers exon 8–9/9, whereas exon 10–11/11 indicates the inclusion of exon 10. **(b)** RPS16 normalized qRT-PCR in paired normal and tumor HNC patients samples using the indicated exon junction specific primers for PKM gene (*n* = 10). **(d)** MeDIP in paired normal and tumor HNC patients samples and qRT-PCR of exon 10 region in PKM gene relative to input (n = 4). **(e-f)** ChIP analysis in paired normal and tumor tissues of HNC patients using (c) RNA Pol II and (d) BORIS antibody, followed by qRT-PCR relative to input (n = 3). Graphs show mean values ± SD. *P* as calculated using two-tailed Student’s t-test,*P < 0.05, ** *P* < 0.01, *** *P* < 0.001, ns = non-significant

The PKM2 isoform has been reported to be upregulated in various cancers [2, 5]. Here we analyzed the HNC profiles available in the Oncomine database [28] and found the overexpression of PKM2 (Additional file 1a-c) in tumor tissue as compared to normal tissue obtained from the patients with HNC. We validated the expression of *PKM* isoforms in the tissue samples obtained from HNC patients under treatment at the Bansal Hospital, Bhopal and observed the higher PKM2 and low PKM1 expression at RNA level (Fig. 1b) by performing the qRT PCR using the isoform-specific exon junction primers (Fig. 1a) as well as at the protein level in all the HNC tissues as compared with the paired normal (Additional file 1f). Earlier, we and others have described the role of intragenic DNA methylation in alternative splicing of various genes [14, 29, 30]. To examine the role of DNA methylation in the regulation of *PKM* splicing, we performed methylated DNA immunoprecipitation (MeDIP) using an antibody specific for 5-methylcytosine.

Interestingly, we observed the high methylation-level at exon-10 (Fig. 1c) whereas no change in methylation was observed at exon-9 and 11 (Additional file 1g). The higher DNA methylation at exon-10 of *PKM*-gene correlates with the inclusion of exon-10 in tumor tissue compared with the paired normal. Intragenic DNA-methylation has been reported to regulate the recruitment of methyl-dependent DNA binding proteins such as BORIS or CTCF [14, 29]. Although CTCF binding site is present at PKM exon-10, the DNA methylation inhibits the binding of CTCF while its paralog BORIS preferentially binds with the methylated-DNA. We hypothesized that the DNA methylation at exon-10 might be favoring the preferential expression of the PKM2-isoform by regulating the binding of a methyl-sensitive DNA binding protein BORIS. BORIS usually is expressed in primary spermatocytes, but it is known to be overexpressed in cancer cells [31]. Next, we analyzed the HNC cancer profiles available in the Oncomine database [28] and observed the positive correlation of BORIS expression (Additional file 1d-e) with the HNC cancer. We also observed the BORIS over-expression at protein level in HNC patients samples (Additional file 1f). Then we performed the BORIS-ChIP to check whether the BORIS binds at exon-10. We observed the BORIS enrichment at PKM exon-10 (Fig. 1d) and no change at exon-9 and 11 (Additional file 1 h) in HNC tumor tissue compared with the paired normal. This observation of BORIS enrichment at exon-10 correlates with the higher DNA-methylation at exon-10 as well as the inclusion of exon-10. We further investigated whether this DNA methylation-mediated binding of BORIS promotes the inclusion of exon-10 by interfering the RNA pol II elongation rate as the hindrance in the RNA pol II elongation rate has been reported to affect the alternative splicing [32, 33]. RNA pol II chip confirmed significantly enriched RNA pol II at PKM exon-10 (Fig. 1e) using the exon-specific primers (Fig. 1a) in HNC patients samples while no change at exon-9 and 11 (Additional file 1i). Together, these observations in clinical samples explain the role of DNA methylation-mediated recruitment of BORIS in *PKM* splicing.

### Treatment with curcumin nanoformulation efficiently leads to the reduction of the tumor-specific isoform of *PKM* gene in HNC H157 cell lines by affecting intragenic DNA methylation

Considering the role of curcumin in modulating the Warburg-effect [19] and DNA methylation [21, 22], we investigated whether curcumin-mediated inhibition of Warburg-effect is dependent on its role in regulation of *PKM* splicing. One of the limitations of using curcumin is its bioavailability [34]. To overcome this limitation, curcumin-loaded amphiphilic polyaspartamide polyelectrolytes-complexes (PECs) were prepared and achieved enhanced nuclear transport of curcumin delivery inside cancer cells [27] (Additional file 2a). Firstly, we examined the effects of curcumin-loaded PEC (curcumin-PEC) as well as curcumin dissolved in ethanol (free-curcumin) on the cell viability of H157 HNC cells. The cells were treated with different concentrations of curcumin-PECs and free-curcumin over a range from 5 μM to 75 μM for 24 h and cell-viability of H157 HNC cells was inspected by trypan-blue assay. We observed the inhibitory concentration (IC_50_) value at 60 μM for curcumin-PECs and observed less toxicity by curcumin-PECs in comparison with the free-curcumin (Additional file 2b). The increased toxicity of free-curcumin as compared to curcumin-PECs was found to be due to the solvent in which free-curcumin was dissolved as shown in (Additional file 2c) and PECs were found to be less toxic. Next, we screened the effect of curcumin-PECs as well as free-curcumin on *PKM* splicing using different concentrations (from 5 μM to 40 μM) using two HNC cell lines (H413 and H157) and an increased switch in *PKM* alternative splicing achieved by Curcumin-PECs (Additional file 2f) as compared to the free-curcumin (Additional file 2 g) which was more prominent in H157 as compared to H413 with an optimal concentration observed at 25 μM in 48 h treatment (Additional file 2f-g). Hence, based on the observation of PKM splicing switch we performed all other experiments in H157 cells.

To understand the reason for better effect on *PKM* splicing by curcumin-PECs as compared to free-curcumin, we measured the curcumin-uptake at 2.5μM as well as at 25μM and observed that the curcumin-uptake was higher with curcumin-PEC as compared to free-curcumin at 2.5μM but there was no significant difference in the curcumin-uptake at 25μM (Additional file 2d). Subsequently, we assessed the retention-efficiency of curcumin at different time-points and observed that curcumin-PECs retention is significantly higher at 12-24h time-points as compared to the free-curcumin (Additional file 2e).

**Fig. 2:**
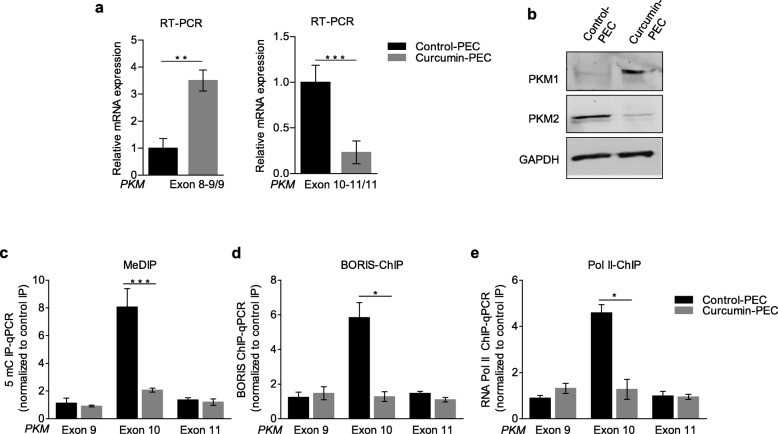
Effect of curcumin treatment on splicing of PKM gene. **(a)** RPS16 normalized qRT-PCR in curcumin-PEC treated versus control-PEC using the indicated primers. **(b)** Western blot showing the protein level of PKM1 and PKM2 in curcumin-PEC and control-PEC treated HNC cells, GAPDH act as a loading control. **(c)** MeDIP in curcumin-PEC versus control-PEC in H157 cells and qRT-PCR relative to input. **(d-e)** ChIP in H157 cells treated with curcumin-PEC versus control-PEC using **(d)** BORIS and **(e)** RNA Pol II antibody, followed by qRT-PCR relative to input and normalized to RPS16. Three independent experiments were conducted with mean values ± SD. *P-*value calculated using two-tailed Student’s t-test, * *P* < 0.05, ** *P* < 0.01, *** *P* < 0.001, ns = non-significant

Next, we used the optimal concentration observed with curcumin-PECs (25μM for 48h with the repeated treatment every 24h) for our further experiments. Interestingly, we found that 25μM curcumin-PECs treatment leads to a significant switch in the *PKM* splicing from cancer-specific PKM2 to normal PKM1-isoform both at the mRNA level (Fig. 2a) and protein level (Fig. 2b). In order to investigate whether the observed effect on *PKM* splicing by curcumin-PEC is mediated by modulation of DNA-methylation, we carried out the MeDIP using an antibody specific to 5-mC and found the reduced DNA-methylation at PKM exon-10 in H157 cells treated with curcumin-PECs as compared to the control-PEC cells (Fig. 2c). Considering the role of DNA methylation in BORIS and Pol II enrichment at the exon-10, subsequently, we performed BORIS and RNA Pol II ChIP and observed the reduced BORIS enrichment (Fig. 2d) together with the decreased RNA Pol II occupancy (Fig. 2e) at PKM exon-10 in curcumin-PECs treated cells as compared to control cells. These observations in HNC cells lead us to believe that the curcumin treatment affects DNA-methylation at exon-10, which leads to the reduced BORIS enrichment and RNA pol II occupancy, consequently leading to reduced exon-10 inclusion (Additional file 2h).

### Curcumin treatment inhibits the activity of DNMT3B that results in the reduced expression of cancer-specific PKM2 isoform

**Fig. 3:**
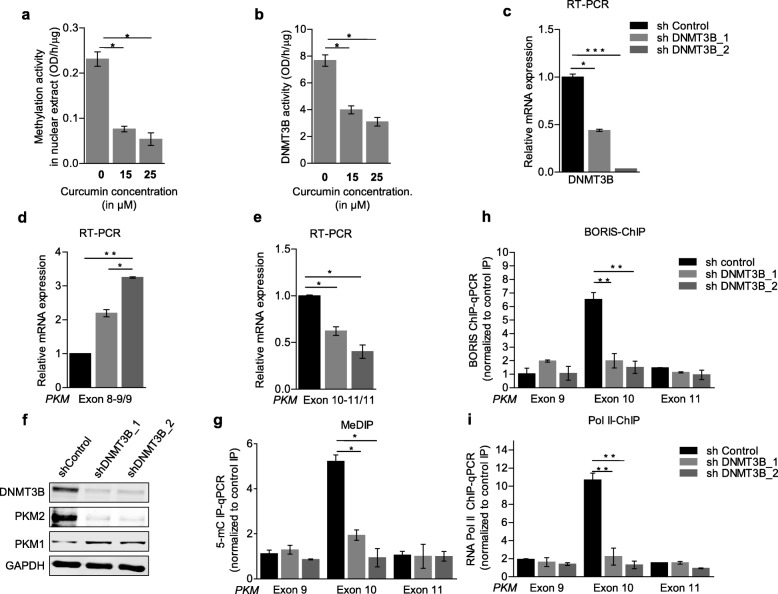
Effect of curcumin on DNMT3B and role of DNMT3B in PKM splicing. **(a-b)** Methyltransferase inhibition activity of curcumin using an in-vitro methyltransferase-assay kit, with **(a)** nuclear-extracts of the HNC cells, **(b)** purified DNMT3B enzyme and **(c-e)** RPS16 normalized qRT-PCR in shDNMT3B transfected cells versus shcontrol using the indicated primers for **(c)** DNMT3B and **(d-e)** PKM gene. **(f)** Western blot showing the protein level of DNMT3B, PKM2, and PKM1 in shDNMT3B transfected cells versus shControl in H157 cells, GAPDH act as a loading control. **(g)** MeDIP in shDNMT3B transfected cells versus shcontrol in H157 cells and qRT-PCR relative to input and control IgG. **(h**-**i)** ChIP in H157 cells transfected with shDNMT3B versus shcontrol using **(h)** BORIS and **(i)** RNA Pol II antibody, followed by qRT-PCR relative to input. Three independent experiments were conducted with mean values ± SD. *P* value using two-tailed Student’s t-test, * *P* < 0.05, ** *P* < 0.01, *** *P* < 0.001, ns = non-significant

**Fig. 4:**
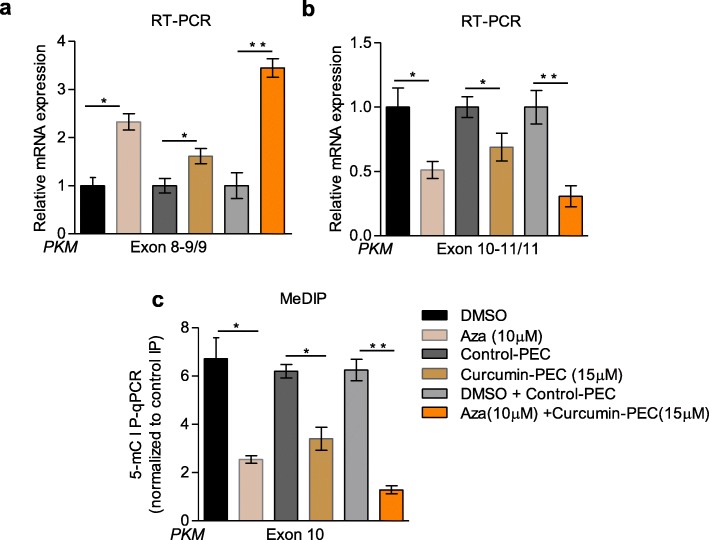
Increased efficacy with combined treatment of 5-Aza-2′-deoxycytidine and curcumin on alternative splicing. **(a-b)** RPS16 normalized qRT-PCR HNC cells treated with Aza + curcumin combination for 48 h using the indicated primers. **(c)** MeDIP in HNC cells treated with Aza + curcumin combination for 48 h and qRT-PCR relative to input and control IgG. Three independent experiments were conducted with mean values ± SD. *P-*value calculated using two-tailed Student’s t-test, * *P* < 0.05, ** *P* < 0.01, *** *P* < 0.001, ns = non significant

Having shown the correlation between DNA methylation and *PKM* alternative splicing, we downregulated the maintenance DNA-methyltransferase 1 (DNMT1) (Additional file 3a) as well as denovo DNA-methyltransferase 3A (DNMT3A) (Additional file 3c) and DNMT3B (Fig. 3c) and observed that there was no significant change in alternative splicing of *PKM* upon DNMT1 (Additional file 3b) and DNMT3A downregulation (Additional file 3d). Notably, downregulation of DNMT3B resulted in reduced DNA-methylation at exon-10 (Fig. 3g) leading to reduced BORIS (Fig. 3h) and Pol II occupancy (Fig. 3i) and thereby exon-10 exclusion (Fig. 3e-f), which is consistent with the previous report on role of DNMT3B in DNA-methylation at PKM exon-10 [14]. Having shown the effect of curcumin-PECs on DNA-methylation and *PKM*-splicing, we investigated the role of curcumin-PECs on DNMT3B expression. We did not observe significant changes in DNMT3B expression upon curcumin-PECs treatment (Additional file 3e), but interestingly, we observed reduced methylation activity of nuclear extract treated with curcumin in an in-vitro experiment (Fig. 3a and Additional file 3f). Moreover, we could see that the purified DNMT3B activity was also inhibited by curcumin as shown in (Fig. 3b and Additional file 3 g), which suggests that the curcumin mediated splicing switch is controlled by its inhibitory effect on DNMT3B activity. As 5-Aza 2′-deoxycytidine (Aza) is a known DNA methylation inhibitor [35], treatment of HNC cells with curcumin and 5-Aza 2′-deoxycytidine (Aza) showed an additive effect on exon-10 DNA-methylation (Fig. 4c) as well as on *PKM*-splicing (Fig. 4a-b, and Additional file 4a-d). Collectively, these results showed the role of curcumin in modulating the DNMT3B activity, leading to reduced DNA methylation as well as the decrease in BORIS and RNA Pol II occupancy at exon-10 and thereby exon-10 exclusion and thus associated with increased expression of normal PKM1 spliced-isoform.

### Curcumin-mediated suppression of Warburg effect and growth inhibition can be rescued by PKM2 overexpression

**Fig. 5:**
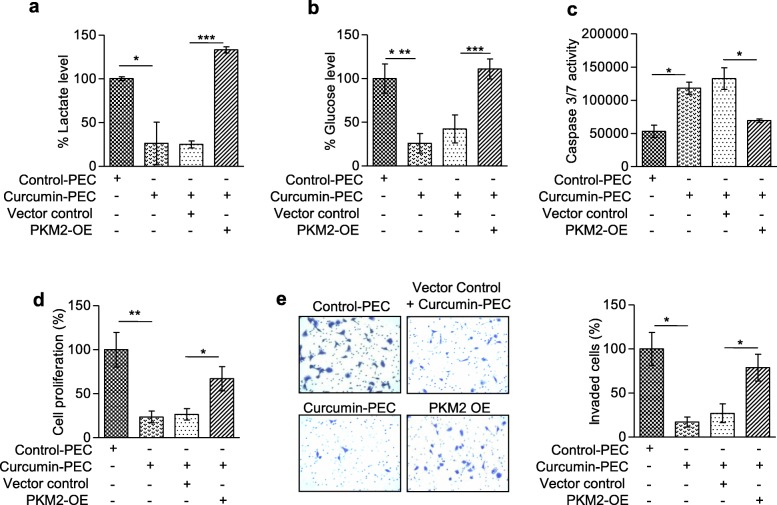
Effect of PKM2 in the maintenance of the Warburg effect in HNC cells.**(a-b)** Percentage of decreased lactate production and glucose uptake in curcumin-PEC treated versus PEC control H157 cells after 48 h of treatment. Decreased lactate production and glucose uptake were rescued after PKM2 over-expression (PKM2-OE) in curcumin-PEC treated cells. **(c)** Measurement of apoptotic death by caspase 3/7 activity in curcumin-PEC treated versus control-PEC H157 cells after 48 h. The apoptotic death was partially rescued after PKM2 over-expression (PKM2-OE) in curcumin-PEC treated cells. **(d)** Reduced cell proliferation in curcumin-PEC treated H157 cell rescued partially by PKM2 overexpression (PKM2-OE) in curcumin-PEC treated H157 cells. **(e)** Representative image of invasion assay in curcumin-PEC treated versus control-PEC H157 cells after 24 h. Over-expression of PKM2 (PKM2-OE) partially rescues the invasion property of curcumin-PEC treated cells and the quantitative evaluation of invasive property of H157 cells. Three independent experiments were conducted with mean values ± SD. *P-*value calculated using two-tailed Student’s t-test, * *P* < 0.05, ** *P* < 0.01,*** *P* < 0.001, ns = non-significant

The overexpression of *PKM2* isoform is associated with the increased Warburg effect [6], and an increase in lactate-production and glucose-uptake are known indicators of increased Warburg effect [2], we examined the effect of curcumin on PKM2-mediated Warburg effect. Notably, curcumin-PECs treatment resulted in lower lactate-production and lower glucose-uptake in HNC cells (Fig. 5a-b). As the observed effect of curcumin-PECs on glucose-uptake and lactate-production is expected to be due to splicing switch from PKM2 to PKM1-isoform, we overexpressed PKM2 in curcumin-PECs treated cells (Additional file 4e). Interestingly, PKM2 over-expression is able to rescue the lactate-production, and glucose-uptake in curcumin-PECs treated cells (Fig.5a-b). As Warburg-effect is associated with increased cell proliferation [2], reduced apoptosis [36] and increased cell invasion [37], we observed the reduction of cell proliferation (Fig.5d) and cell invasion (Fig. 5e), and an increase in apoptosis (Fig.5c) in curcumin-PECs treated cells, which was rescued by PKM2 overexpression. These observations suggest that the known anti-tumor activity of curcumin [38, 39] may partially be explained by its effect on PKM splicing-switch and thereby inhibition of Warburg effect and growth of HNC cells.

### Global effect of curcumin treatment on alternative splicing in HNC cells

Together our data suggest that curcumin plays a significant role in regulating the alternative pre-mRNA splicing of the *PKM* gene by modulating the DNA-methylation-dependent BORIS recruitment. Next, we examined the global changes in alternative pre-mRNA splicing in curcumin-PECs treated HNC cells as compared to the control-PEC cells using the Human Transcriptome Array 2.0 (HTA 2.0) (Additional file 5b). Curcumin-PECs treatment results in the differential alternative splicing of 641 genes (Additional file 5c). Interestingly, the gene ontology analysis of curcumin-mediated alternatively spliced events showed the association of these alternatively spliced genes with various cellular processes such as cell-adhesion, cell-cycle, mRNA-processing and extra-cellular-matrix-organization (Additional file 5d). These alternatively spliced genes were also correlated with tobacco use disorders and head and neck neoplasm (Additional file 6e). This suggests that curcumin controls the alternative splicing of genes involved in major hallmarks of cancer.

**Fig. 6:**
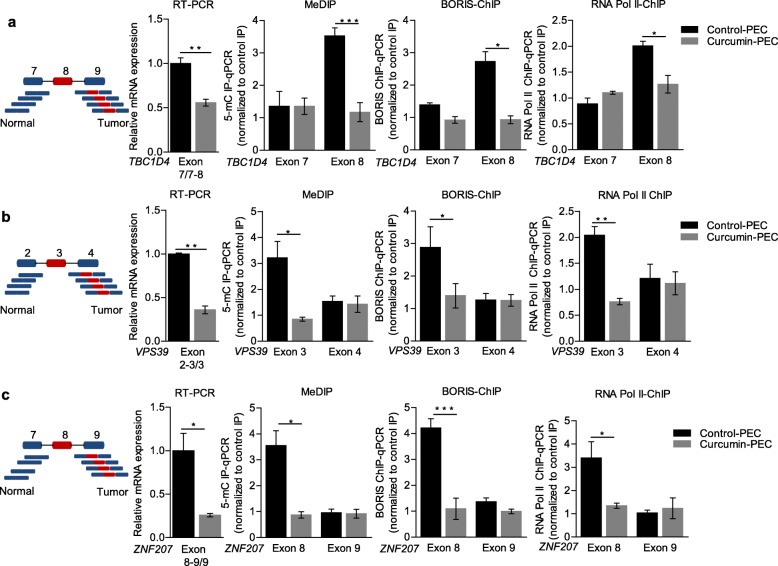
Effect of curcumin on global alternative splicing events. **(a-c)** Schematic representation of **(a)**TBC1D4, **(b)** VPS39, **(c)** ZNF207 and their cancer-specific spliced isoform. RPS16 normalized qRT-PCR in curcumin-PEC treated and control-PEC in H157 cells using the indicated exon junction specific primers for the respective genes. MeDIP in curcumin-PEC treated and control-PEC in H157 cells and qRT-PCR in respective genes relative to input. ChIP in curcumin-PEC treated and PEC-control H157 cells using RNA Pol II and BORIS antibody, followed by qRT-PCR relative to input. Three independent experiments were conducted with mean values ± SD. *P-*value calculated using two-tailed Student’s t-test, * *P* < 0.05, ** *P* < 0.01, *** *P* < 0.001, ns = non-significant

Additionally, we selected a few candidate genes from HTA2.0 array analysis such as (TBC1 Domain Family Member 4) TBC1D4 (Fig. 6a), (Vacuolar Protein Sorting 39 Homolog) VPS39 (Fig. 6b) and (Zinc Finger Protein 207) ZNF207 (Fig. 6c) and validated the change in alternative splicing upon curcumin-PECs treatment as shown in (Fig. 6a-c).

Furthermore, we also observed reduced DNA-methylation (Fig. 6a-c), and decreased BORIS (Fig. 6a-c) and Pol II occupancy (Fig. 6a-c) at the alternative exons of VPS39, ZNF207 and TBC1D4 upon curcumin-PECs treatment leading to the exclusion of the respective alternative exons, suggesting that the curcumin-mediated alternative splicing is not limited to PKM.

## Discussion

Here in this study, we report the underlying epigenetic mechanism of *PKM* alternative splicing in head-and-neck cancer (HNC). Though both epigenetic alterations [40, 41] and aberrant alternative splicing [42, 43] are individually associated with the development and progression of various cancers and epigenetic regulation of alternative splicing in various model systems including lymphocyte development [29], neuronal differentiation [30] and embryonic stem cells [44] is reported, the role of epigenetic alterations in aberrant alternative splicing in cancer cells is not well understood. The deregulation of DNA-methylation is universally associated with various cancers [45], and we have earlier shown the role of DNA methylation-mediated CTCF recruitment in the regulation of CD45 alternative splicing in lymphocyte development [29]. Moreover, DNA-methylation has also been shown to regulate alternative splicing through modulation of methyl-sensitive DNA binding proteins such as MeCP2 [30] and BORIS [14]. Here we have demonstrated that *PKM* splicing-switch is epigenetically regulated by DNA methylation-dependent recruitment of BORIS at exon-10 of *PKM* gene which leads to the inclusion of exon-10 and favors the PKM2 splice-isoform in head-and-neck cancer cells. This observation is consistent with our previous report where we have shown the DNA-methylation dependent recruitment of BORIS and its role in exon-10 inclusion in breast cancer [14]. PKM2-isoform has been found to be over-expressed in cancer cells [5] and is a key regulator of the Warburg effect and thereby cancer metabolism [6]. Hence, targeting PKM2 splicing could be an effective anti-cancer strategy [4].

Interestingly, the epigenetic modifications, including DNA-methylation are potentially reversible and could be targeted to revert the splicing-switch. Here in this study, we present the first mechanistic evidence of curcumin causing the switch in the *PKM* splicing from cancer-specific PKM2-isoform to normal PKM1-isoform by inhibiting the DNMT3B in HNC cells. Our results reveal that curcumin treatment reduces DNA-methylation at exon-10, resulting in the decrease of BORIS and RNA Pol II occupancy, leading to the exon-10 exclusion and thereby increased expression of PKM1 spliced-isoform. Low-bioavailability of Curcumin is a major limitation in its therapeutic application [34]. It has been earlier reported that bioavailability of curcumin is significantly enhanced by using curcumin-nanoformulation prepared using amphiphilic polyaspartamide polyelectrolytes-complexes (PECs). The Curcumin-loaded PECs are readily internalized by a panel of mammalian cells (HEK-293 T, MDA-MB-231, and U2OS). Additionally, these PECs have been shown to significantly improve nuclear transport of curcumin in comparison with free-curcumin [27].

In the present study, to improve the curcumin uptake, we used curcumin-PECs to treat the head and neck cancer cells. Notably, the observed effect of curcumin on *PKM* splicing-switch also results in the inhibition of Warburg effect in terms of reduced glucose-uptake and lactate production and thereby reduced growth, invasion and increased apoptosis of head-and-neck cancer cells. The observed effect of curcumin on alternative splicing of *PKM* gene together with its inhibitory effect on the growth of HNC cancer cells is coherent with the previously studied anti-cancer activity of curcumin in various cancer [24] and thus categorize curcumin as a promising anti-cancer therapeutic drug.

Next, the global changes were observed in alternative pre-mRNA splicing in curcumin-PECs treated HNC cells as compared to the control cells using the Human Transcriptome Array 2.0 (HTA 2.0). We validated the splicing of few candidate genes such as (TBC1 Domain Family Member 4) TBC1D4, (Vacuolar Protein Sorting 39 Homolog) VPS39, and (Zinc Finger Protein 207) ZNF207 and observed that the DNA methylation-dependent splicing is not limited to the *PKM* gene. The VPS39 is a critical subunit of the homotypic fusion and vacuole protein sorting (HOPS) late endosomal complex and plays an important role in the cellular invasion in breast cancer [46]. Similarly, ZNF207 is a kinetochore component protein, which plays an essential role in spindle assembly and is associated with the progression of glioblastoma multiforme [47], while TBC1D4 has been shown to have its role in GLUT4-cell membrane translocation and has been positively correlated with Akt-pathway in non-small cell lung carcinoma [48]. These results highlight two important points, first being the curcumin treatment results in the genome-wide changes in alternative splicing in HNC cells and second is the effect of curcumin on DNA-methylation dependent alternative splicing is not limited to PKM. This is the first study which shows the role of DNA-methylation mediated BORIS recruitment in alternative splicing of TBC1D4, VPS39, and ZNF207.

Furthermore, we have shown that the curcumin treatment results in the splicing-switch in a DNA methylation-dependent manner. Collectively, the splicing modulation of VPS39, ZNF207, and TBC1D4 in curcumin-treated HNC cells provides the basis for future investigation of the role of cancer-specific isoforms of these genes in tumorigenesis. Finally, our gene-ontology analysis of curcumin-mediated alternatively spliced events showed the association of shortlisted alternatively spliced genes with various cellular processes involved in tumor progression, which suggests that, curcumin may target alternative splicing of several genes associated with the hallmarks of cancer.

Collectively, we have demonstrated the role of upstream DNA-methylation in the regulation of BORIS recruitment and thereby exon-10 inclusion leading to PKM2-isoform expression in HNC cells. The PKM2 overexpression results in increased Warburg effect and supports the growth of cancer cells. Moreover, this study provides the mechanism to switch the *PKM* splicing from cancer-specific PKM2-isoform to normal PKM1-isoform, leading to reduced Warburg effect and reduced growth of HNC cells by use of curcumin, a nutraceutical. Consequently, our study highlights the potential of nutraceuticals in reversion of cancer-specific splicing and thereby may provide avenues for therapeutic management of head and neck cancer in the future.

## Conclusion

Taken together, we have identified DNA methylation-mediated mechanism of PKM alternative splicing to support the increased expression of PKM2 in HNC and reports that the curcumin treatment can revert the splicing switch from cancer-specific PKM2 to normal PKM1 isoform, resulting in reduced Warburg effect. Our finding provides an effective way to modulate cancer-specific-splicing and thereby could be of significant clinical relevance as the administration of curcumin might improve the therapeutic outcome of HNC.

## Supplementary information

**Additional file 1: Fig. S1.** PKM protein expression and DNA methylation in HNC patients(a-c) PKM2 expression in HNC tumor samples extracted from (a) Cromer Head-Neck, (b) Toruner Head-Neck and (c) Ye Head-Neck cancer analyzed by using Oncomine database. (d-e) shows the stage-wise expression of BORIS in (d) Ye Head-Neck and (e) Slebos Head-Neck cancer analyzed by using Oncomine database. (f) Western blot showing the protein level of PKM2, PKM1, and BORIS in paired normal and tumor tissue of HNC patients, GAPDH act as a loading control. (g) MeDIP in paired normal and tumor tissue of HNC patients samples and qRT-PCR of PKM exon 9 and exon 11 region, relative to input and control IgG (*n* = 4). (h-i) ChIP in paired normal and tumor tissue of HNC patients, (i) using RNA Pol II and (h) BORIS antibody and qRT-PCR with indicated exonic primers relative to input and control IgG(*n* = 3). Graphs show mean values ± SD. *P-*value calculated using two-tailed Student’s t-test, * *P* < 0.05, ** *P* < 0.01, *** *P* < 0.001, ns = non-significant.

**Additional file 2: Fig. S2.** Relative curcumin uptake: (a) Diagrammatic representation of curcumin nanoparticle formulation. Amphiphilic polyelectrolytes (PEs) allow efficient entrapment of the hydrophobic drug, Curcumin. The drug-loaded PECs achieve intracellular delivery of Curcumin, resulting in efficient entry of curcumin into the cells. (b-c) Cell-viability assays by trypan blue method (b) in HNC cells after treatment for 24 h with curcumin-PEC and free-curcumin, (c) MTT assay of H157 cells after treatment with different concentration of DMSO, Ethanol and PEC control at the time point of 24 h. (d) Fluorescence microscopic images for free-curcumin and curcumin-PEC treated H157 cells, (e) Relative percent curcumin retention in H157 cells after treatment with free-curcumin and curcumin-PEC, (f-g) RPS16 normalized qRT-PCR in HNC cells treated with (f) curcumin-PEC versus PEC control and (g) free-curcumin versus ethanol-control using the indicated exon junction specific primers for PKM gene. (h) Semi-q PCR was performed followed by PstI digestion to distinguish the PKM 1 and PKM 2. Three independent experiments were conducted with mean values ± SD. *P* as calculated using two-tailed Student’s t-test, * *P* < 0.05, ** *P* < 0.01, *** *P* < 0.001, ns = non-significant.

**Additional file 3: Fig. S3.** Effect of DNMTs in PKM splicing. (a-b) RPS16 normalized qRT-PCR in shDNMT1 transfected cells versus shcontrol using the indicated primers for (a) DNMT1 and (b) PKM (c-d) RPS16 normalized qRT-PCR in shDNMT3A transfected cells versus shcontrol using the indicated primers for (c) DNMT3A and (d) PKM gene. (e) Western blot showing the protein level of DNMT3B in curcumin-PEC treated versus control PEC, GAPDH act as a loading control. (f-g) Curcumin affected level of methylation activity, in (f) pure DNMT3B enzyme, and (g) nuclear-extract from HNC cells using in vitro methyltransferase kit. Three independent experiments were conducted with mean values ± SD. *P* value calculated using two-tailed Student’s t-test, * *P* < 0.05, ** *P* < 0.01, *** *P* < 0.001, ns = non-significant.

**Additional file 4: Fig. S4.** Combinatorial effect of Curcumin and 5-Aza-2′-deoxycytidine treatment affects PKM splicing. (a-d) RPS16 normalized qRT-PCR in H157 cells upon treatment with different concentration of (a-b) Aza and (c-d) Aza + curcumin for 48 h to check the splicing of PKM gene using indicated exon junction specific primers. (e) Western-blot showing the Flag-tagged PKM2 in vector control and PKM2-overexpression (PKM2-OE) transfected in H157 cells. GAPDH acts as a loading control. Three independent experiments were conducted with mean values ± SD. *P-*value calculated using two-tailed Student’s t-test, * *P* < 0.05, ** *P* < 0.01, *** *P* < 0.001, ns = non-significant.

**Additional file 5: Fig. S5.** Effect of Curcumin on global alternative splicing events. (a) Clustering analysis of top 30 spliced events (15 exclusion and 15 inclusion exons) in curcumin-PEC treated versus control-PEC H157 cells. (a) Pi-graph for global differential alternatively spliced genes. (b) Global differential expression pattern of alternatively spliced exons in curcumin-PEC treated versus control-PEC H157 cell line (*n* = 2 splicing index ≤ − 2 and ≥ + 2; P<0.05). The graph represents normalized expression levels (SST-RMA algorithm) of differentially expressed exon probes. Red color dots represent exon exclusion. (c) Gene Ontology analysis of alternatively spliced genes upon curcumin-PEC treatment in H157 cells. The graph shows the top GO functions regulated in molecular and biological process category. (d) The graph shows the correlation of spliced genes with the disease category. (*n* = 2).

## Data Availability

The HTA 2.0 array data reported in this report have been deposited in the Gene-Expression-Omnibus (GEO) database, www.ncbi.nlm.nih.gov/geo (accession no. GSE126191). All other data produced and obtained is available within the manuscript.

## Abbreviations

Aza 5: Aza 2′-deoxycytidine
BORIS: Brother of Regulator of Imprinted Sites
ChIP: Chromatin ImmunoPrecipitation
DNMT1: DNA Methyltransferase 1
DNMT3A: DNA Methyltransferase 3A
DNMT3B: DNA Methyltransferase 3B
HNC: Head and Neck Cancer
hnRNP: Heterogeneous Nuclear Ribonucleoprotein
HTA 2.0 array: Human Transcriptome Array
MeDIP: Methylated DNA ImmunoPrecipitation
PKM: Pyruvate Kinase Muscle
PTB: Polypyrimidine Tract-Binding protein 1
SRSF3: Serine and Arginine rich Splicing Factor 3
