# Supplementary figures and images for: Dietary-phytochemical mediated reversion of cancer-specific splicing inhibits Warburg effect in head and neck cancer
Source: BMC Cancer. 2019 Nov 1;19:1031. doi: 10.1186/s12885-019-6257-1 (PMC6823945; doi:10.1186/s12885-019-6257-1)

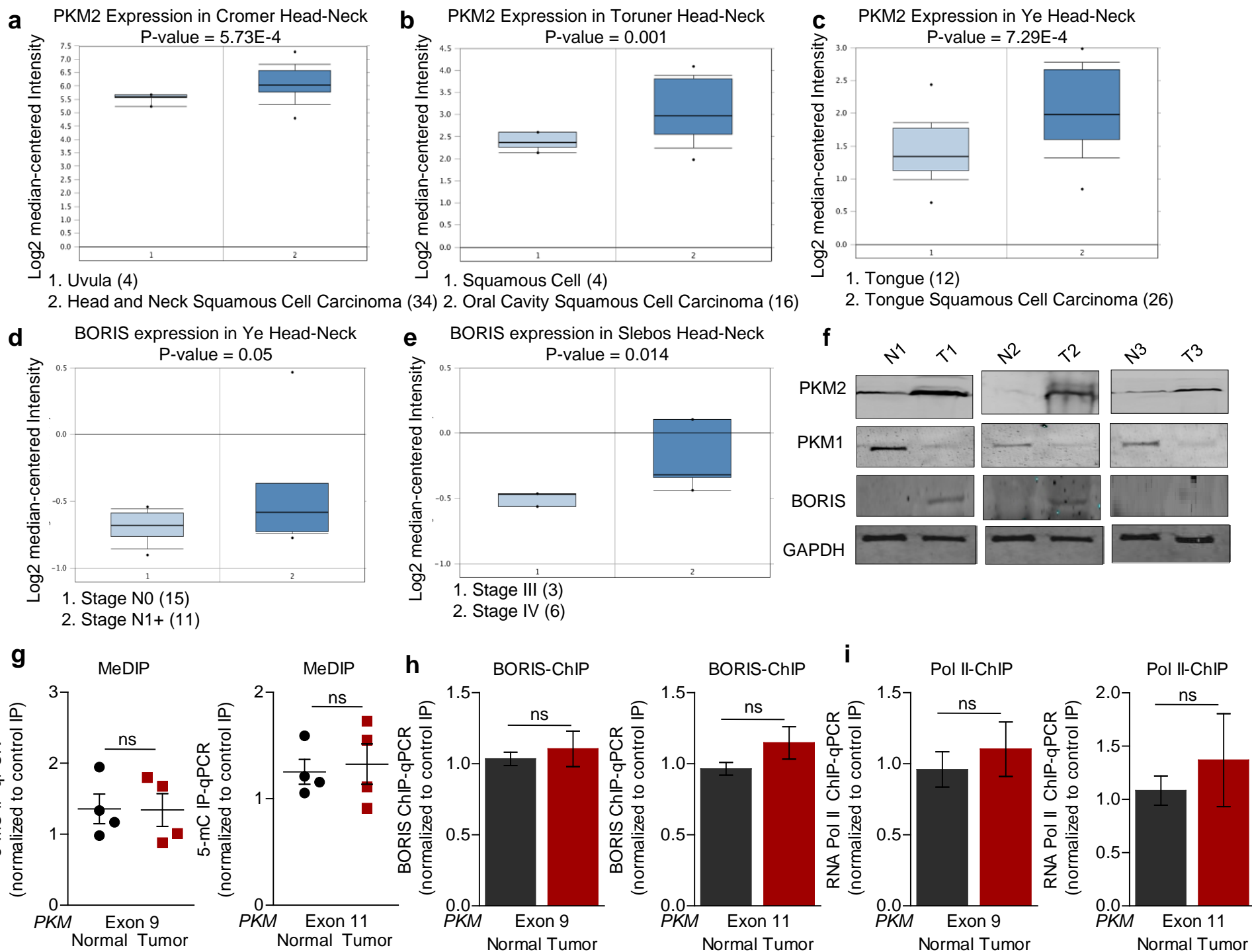

Supplement: Supplementary file 1 — Additional file 1: Fig. S1. PKM protein expression and DNA methylation in HNC patients(a-c) PKM2 expression in HNC tumor samples extracted from (a) Cromer Head-Neck, (b) Toruner Head-Neck and (c) Ye Head-Neck cancer analyzed by using Oncomine database. (d-e) shows the stage-wise expression of BORIS in (d) Ye Head-Neck and (e) Slebos Head-Neck cancer analyzed by using Oncomine database. (f) Western blot showing the protein level of PKM2, PKM1, and BORIS in paired normal and tumor tissue of HNC patients, GAPDH act as a loading control. (g) MeDIP in paired normal and tumor tissue of HNC patients samples and qRT-PCR of PKM exon 9 and exon 11 region, relative to input and control IgG (n = 4). (h-i) ChIP in paired normal and tumor tissue of HNC patients, (i) using RNA Pol II and (h) BORIS antibody and qRT-PCR with indicated exonic primers relative to input and control IgG(n = 3). Graphs show mean values ± SD. P-value calculated using two-tailed Student’s t-test, * P < 0.05, ** P < 0.01, *** P < 0.001, ns = non-significant. [file 12885_2019_6257_MOESM1_ESM.pdf]

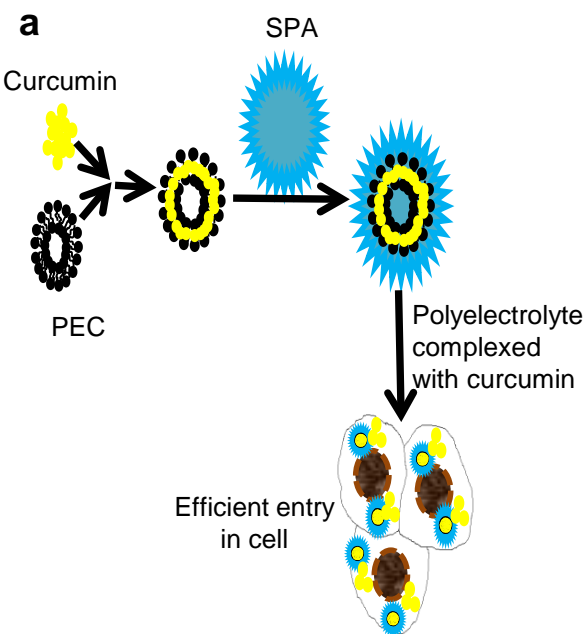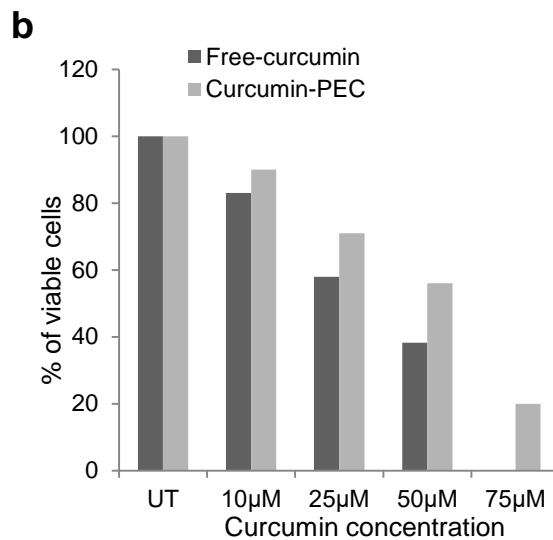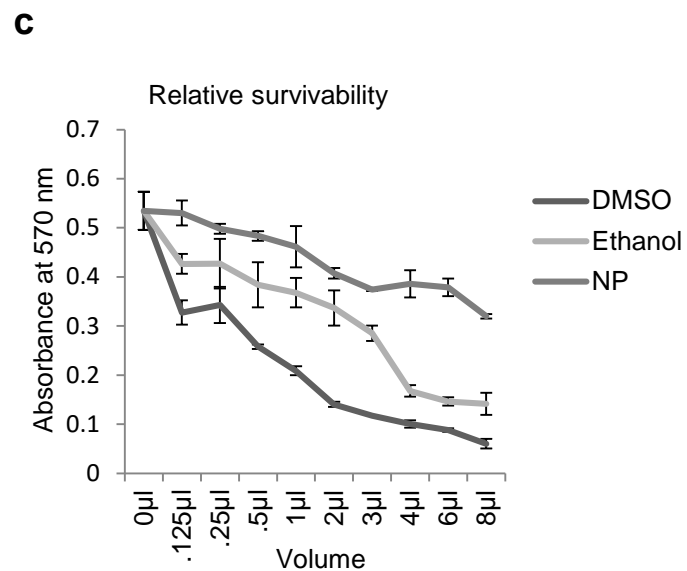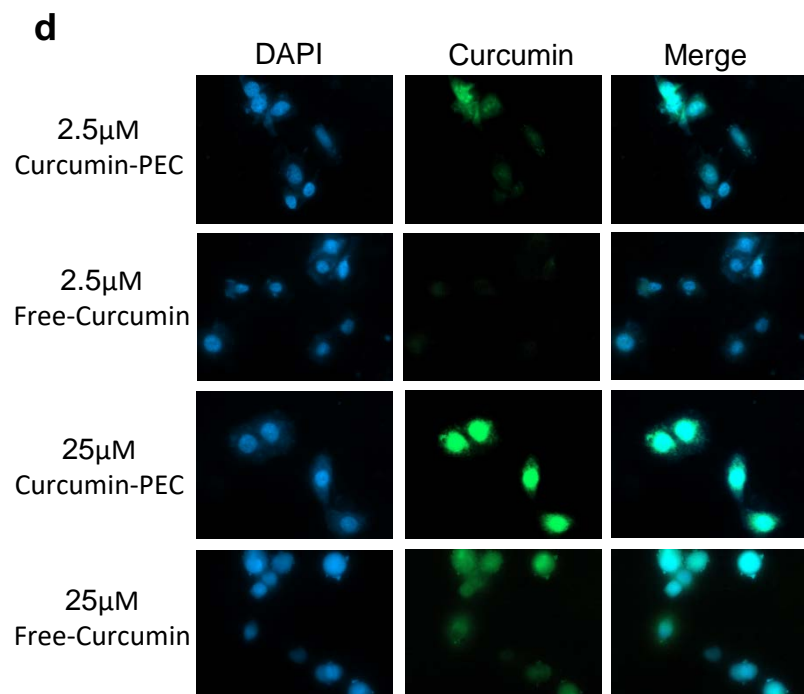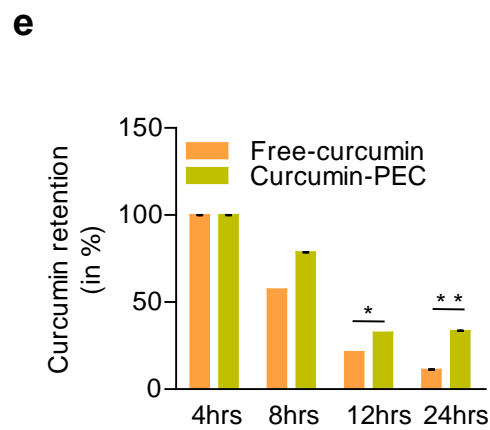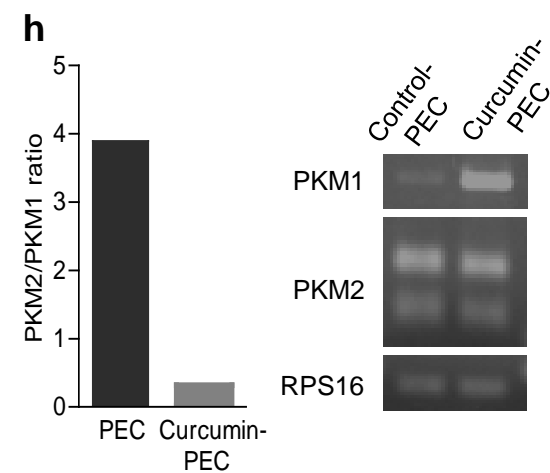

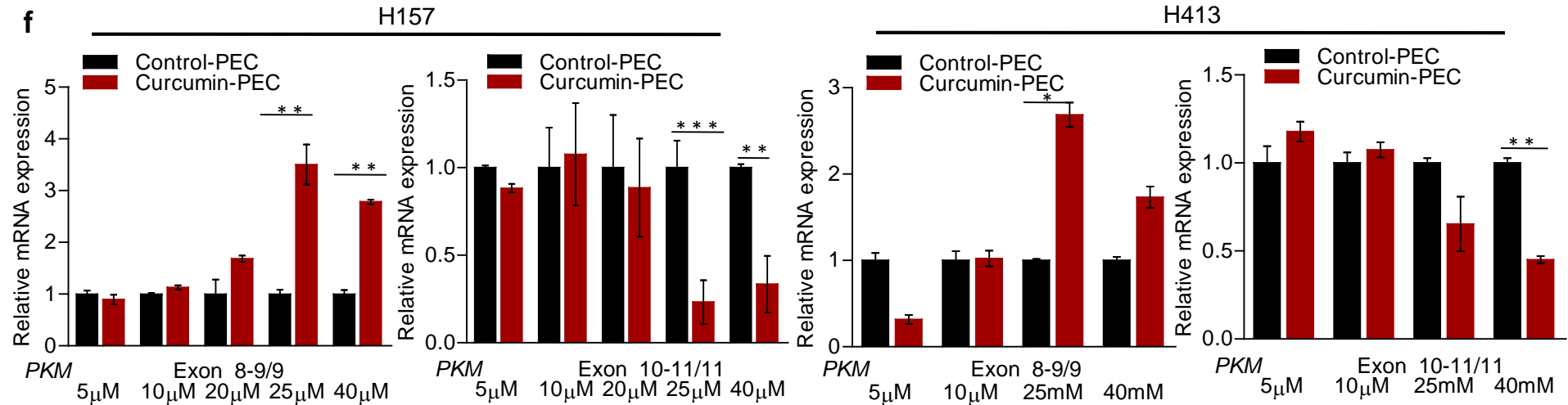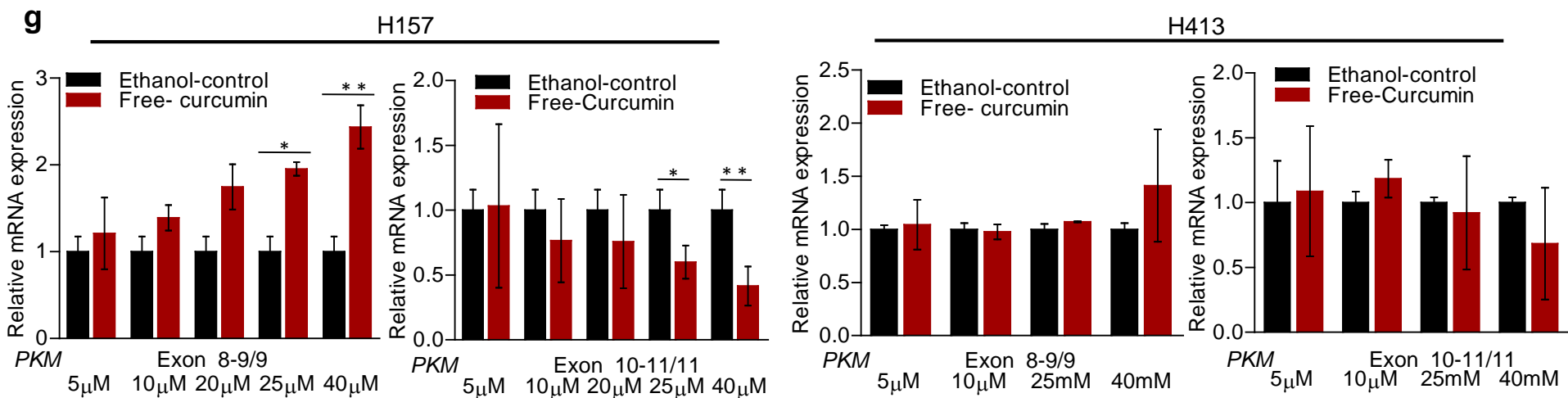

Supplement: Supplementary file 2 — Additional file 2: Fig. S2. Relative curcumin uptake: (a) Diagrammatic representation of curcumin nanoparticle formulation. Amphiphilic polyelectrolytes (PEs) allow efficient entrapment of the hydrophobic drug, Curcumin. The drug-loaded PECs achieve intracellular delivery of Curcumin, resulting in efficient entry of curcumin into the cells. (b-c) Cell-viability assays by trypan blue method (b) in HNC cells after treatment for 24 h with curcumin-PEC and free-curcumin, (c) MTT assay of H157 cells after treatment with different concentration of DMSO, Ethanol and PEC control at the time point of 24 h. (d) Fluorescence microscopic images for free-curcumin and curcumin-PEC treated H157 cells, (e) Relative percent curcumin retention in H157 cells after treatment with free-curcumin and curcumin-PEC, (f-g) RPS16 normalized qRT-PCR in HNC cells treated with (f) curcumin-PEC versus PEC control and (g) free-curcumin versus ethanol-control using the indicated exon junction specific primers for PKM gene. (h) Semi-q PCR was performed followed by PstI digestion to distinguish the PKM 1 and PKM 2. Three independent experiments were conducted with mean values ± SD. P as calculated using two-tailed Student’s t-test, * P < 0.05, ** P < 0.01, *** P < 0.001, ns = non-significant. [file 12885_2019_6257_MOESM2_ESM.pdf]

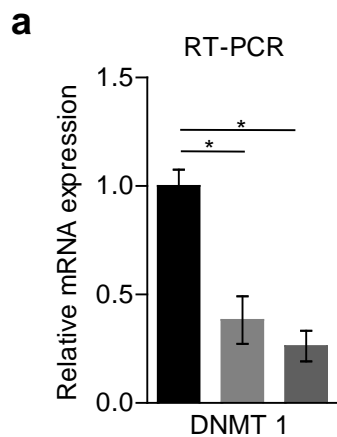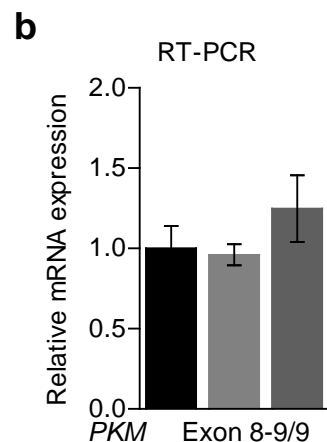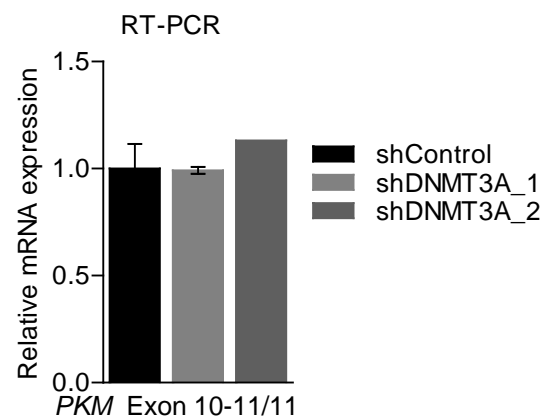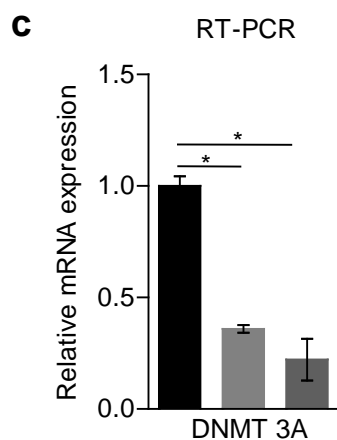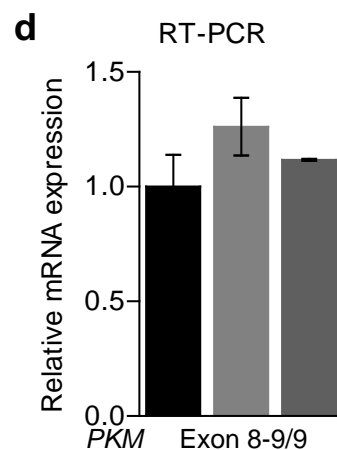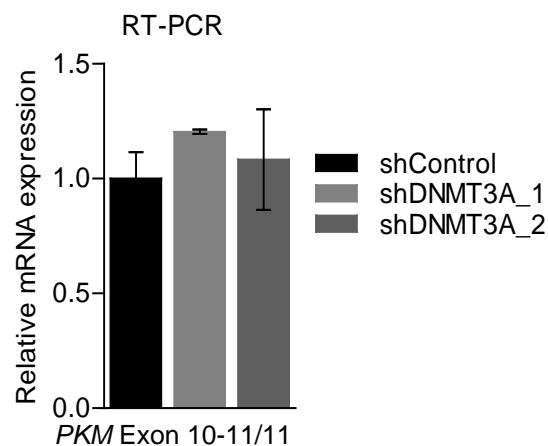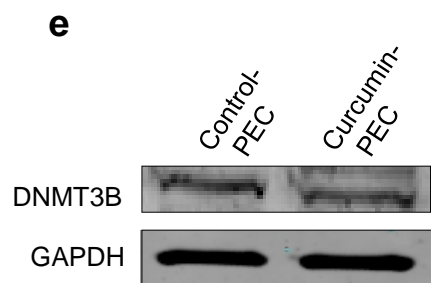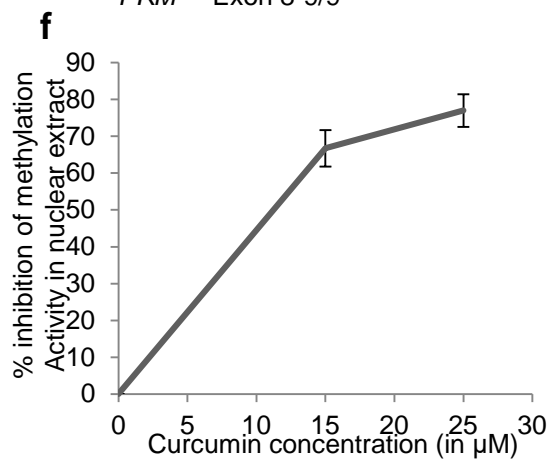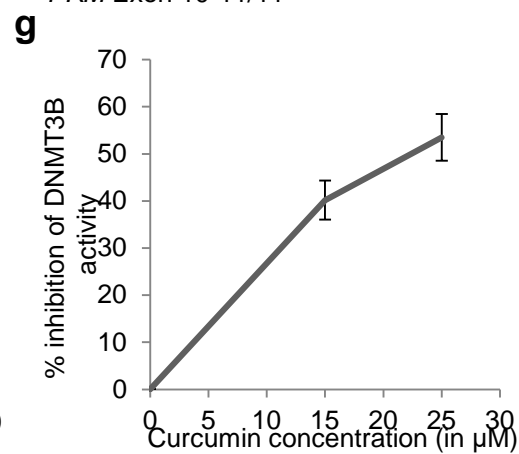

Supplement: Supplementary file 3 — Additional file 3: Fig. S3. Effect of DNMTs in PKM splicing. (a-b) RPS16 normalized qRT-PCR in shDNMT1 transfected cells versus shcontrol using the indicated primers for (a) DNMT1 and (b) PKM (c-d) RPS16 normalized qRT-PCR in shDNMT3A transfected cells versus shcontrol using the indicated primers for (c) DNMT3A and (d) PKM gene. (e) Western blot showing the protein level of DNMT3B in curcumin-PEC treated versus control PEC, GAPDH act as a loading control. (f-g) Curcumin affected level of methylation activity, in (f) pure DNMT3B enzyme, and (g) nuclear-extract from HNC cells using in vitro methyltransferase kit. Three independent experiments were conducted with mean values ± SD. P value calculated using two-tailed Student’s t-test, * P < 0.05, ** P < 0.01, *** P < 0.001, ns = non-significant. [file 12885_2019_6257_MOESM3_ESM.pdf]

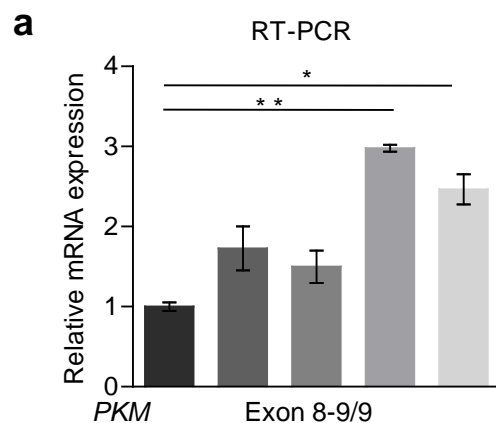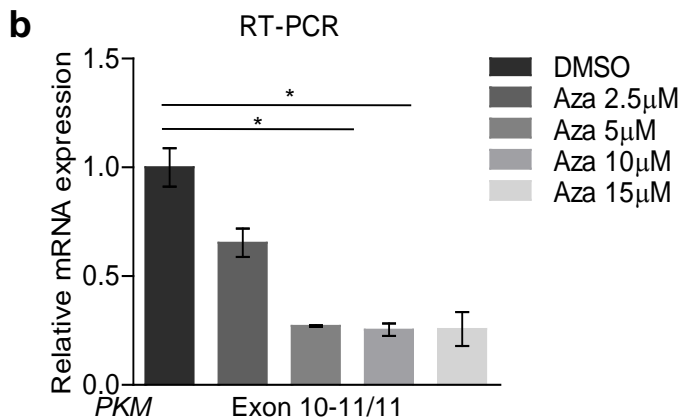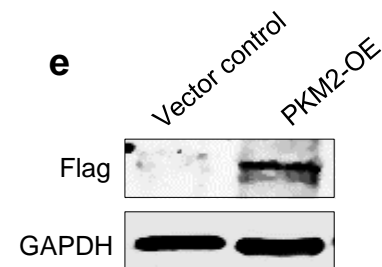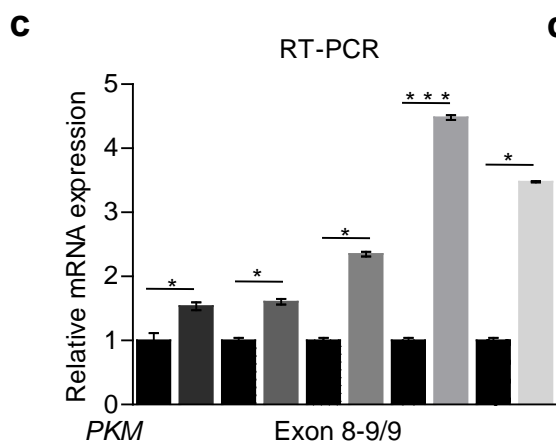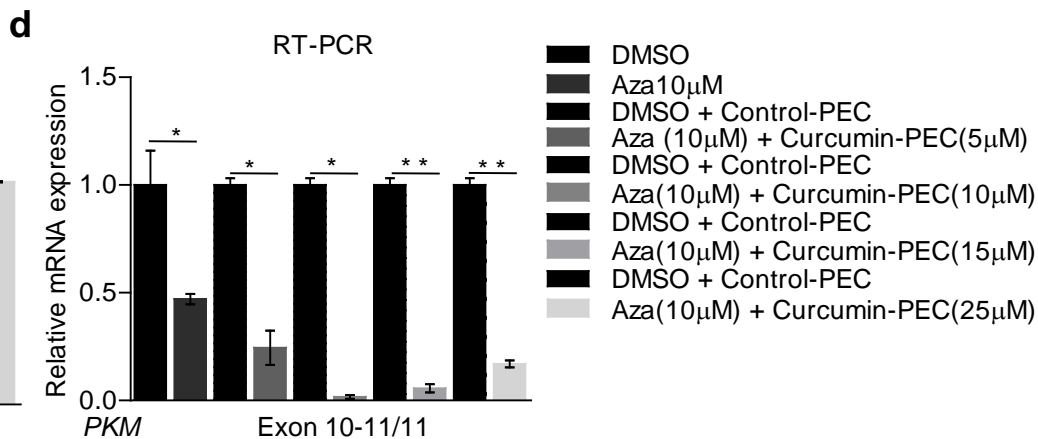

Supplement: Supplementary file 4 — Additional file 4: Fig. S4. Combinatorial effect of Curcumin and 5-Aza-2′-deoxycytidine treatment affects PKM splicing. (a-d) RPS16 normalized qRT-PCR in H157 cells upon treatment with different concentration of (a-b) Aza and (c-d) Aza + curcumin for 48 h to check the splicing of PKM gene using indicated exon junction specific primers. (e) Western-blot showing the Flag-tagged PKM2 in vector control and PKM2-overexpression (PKM2-OE) transfected in H157 cells. GAPDH acts as a loading control. Three independent experiments were conducted with mean values ± SD. P-value calculated using two-tailed Student’s t-test, * P < 0.05, ** P < 0.01, *** P < 0.001, ns = non-significant. [file 12885_2019_6257_MOESM4_ESM.pdf]
