# Supplementary material for: Dietary-phytochemical mediated reversion of cancer-specific splicing inhibits Warburg effect in head and neck cancer
Source: BMC Cancer. 2019 Nov 1;19:1031. doi: 10.1186/s12885-019-6257-1 (PMC6823945; doi:10.1186/s12885-019-6257-1)
Supplement: Supplementary file 5 — Additional file 5: Fig. S5. Effect of Curcumin on global alternative splicing events. (a) Clustering analysis of top 30 spliced events (15 exclusion and 15 inclusion exons) in curcumin-PEC treated versus control-PEC H157 cells. (a) Pi-graph for global differential alternatively spliced genes. (b) Global differential expression pattern of alternatively spliced exons in curcumin-PEC treated versus control-PEC H157 cell line (n = 2 splicing index ≤ − 2 and ≥ + 2; P<0.05). The graph represents normalized expression levels (SST-RMA algorithm) of differentially expressed exon probes. Red color dots represent exon exclusion. (c) Gene Ontology analysis of alternatively spliced genes upon curcumin-PEC treatment in H157 cells. The graph shows the top GO functions regulated in molecular and biological process category. (d) The graph shows the correlation of spliced genes with the disease category. (n = 2). [file 12885_2019_6257_MOESM5_ESM.pdf]

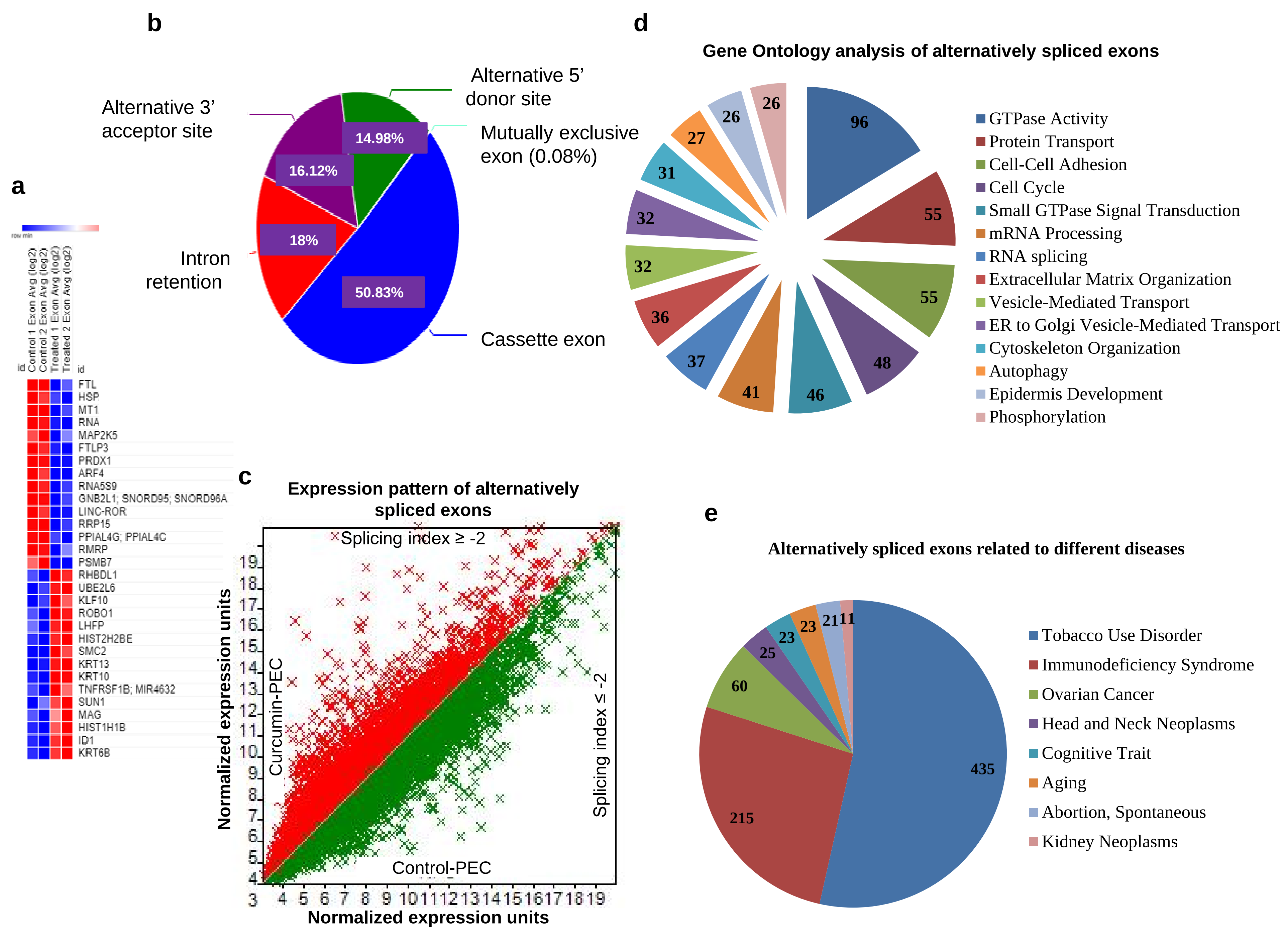

a
row min
id
Control 1 Exon Avg (log2)
Control 2 Exon Avg (log2)
Treated 1 Exon Avg (log2)
Treated 2 Exon Avg (log2)
id
FTL
HSP.
MT1.
RNA
MAP2K5
FTLP3
PRDX1
ARF4
RNA5S9
GNB2L1; SNORD95; SNORD96A
LINC-ROR
RRP15
PPIAL4G; PPIAL4C
RMRP
PSMB7
RHBDL1
UBE2L6
KLF10
ROBO1
LHFP
HIST2H2BE
SMC2
KRT13
KRT10
TNFRSF1B; MIR4632
SUN1
MAG
HIST1H1B
ID1
KRT6B
b
Alternative 3' acceptor site
Alternative 5' donor site
Mutually exclusive exon (0.08%)
Intron retention
Cassette exon
14.98%
16.12%
18%
50.83%
c
Expression pattern of alternatively spliced exons
Splicing index ≥ -2
Splicing index ≤ -2
Curcumin-PEC
Control-PEC
Normalized expression units
Normalized expression units
d
Gene Ontology analysis of alternatively spliced exons
96
55
55
48
46
41
37
36
32
32
31
27
26
26
GTPase Activity
Protein Transport
Cell-Cell Adhesion
Cell Cycle
Small GTPase Signal Transduction
mRNA Processing
RNA splicing
Extracellular Matrix Organization
Vesicle-Mediated Transport
ER to Golgi Vesicle-Mediated Transport
Cytoskeleton Organization
Autophagy
Epidermis Development
Phosphorylation
e
Alternatively spliced exons related to different diseases
435
215
60
25
23
23
21
11
Tobacco Use Disorder
Immunodeficiency Syndrome
Ovarian Cancer
Head and Neck Neoplasms
Cognitive Trait
Aging
Abortion, Spontaneous
Kidney Neoplasms
